# Binocular Vision, Visual Function, and Pupil Dynamics in People Living With Dementia and Their Relation to the Rate of Cognitive Decline and Structural Changes Within the Brain: Protocol for an Observational Study

**DOI:** 10.2196/16089

**Published:** 2020-08-10

**Authors:** Marianne Piano, Ramin Nilforooshan, Simon Evans

**Affiliations:** 1 School of Health Sciences Faculty of Health and Medical Sciences University of Surrey Guildford United Kingdom; 2 Department of Optometry and Vision Sciences University of Melbourne Melbourne Australia; 3 National Vision Research Institute Australian College of Optometry Melbourne Australia; 4 Surrey and Borders Partnership NHS Trust Chertsey United Kingdom; 5 School of Psychology Faculty of Health and Medical Sciences University of Surrey Guildford United Kingdom

**Keywords:** binocular vision, dementia, magnetic resonance imaging, stereopsis, pupil, sleep

## Abstract

**Background:**

Visual impairment is a common comorbidity in people living with dementia. Addressing sources of visual difficulties can have a significant impact on the quality of life for people living with dementia and their caregivers. Depth perception problems are purportedly common in dementia and also contribute to falls, visuomotor task difficulties, and poorer psychosocial well-being. However, depth perception and binocular vision are rarely assessed in dementia research. Sleep fragmentation is also common for people living with dementia, and binocular cooperation for depth perception can be affected by fatigue. Pupillary responses under cognitive load also have the potential to be a risk marker for cognitive decline in people living with dementia and can be combined with the above measures for a comprehensive evaluation of clinical visual changes in people living with dementia and their relation to changes in cognitive status, sleep quality, and cortical structure or function.

**Objective:**

This study aims to characterize the nature of clinical visual changes and altered task-evoked pupillary responses that may occur in people living with dementia and evaluate whether these responses relate to changes in cognitive status (standardized Mini Mental State Examination [MMSE] score), Pittsburgh sleep quality index, and cortical structure or function.

**Methods:**

This proposed exploratory observational study will enroll ≤210 people with recently diagnosed dementia (within the last 24 months). The following parameters will be assessed on 3 occasions, 4 months apart (plus or minus 2 weeks): visual function (visual acuity and contrast sensitivity), binocular function (motor fusion and stereopsis), task-evoked pupillary responses (minimum and maximum pupil size, time to maximum dilation, and dilation velocity), cognitive status (MMSE score), and sleep quality (Pittsburgh Sleep Quality Index). A subset of patients (n=30) with Alzheimer disease will undergo structural and functional magnetic resonance imaging at first and third visits, completing a 10-day consensus sleep diary to monitor sleep quality, verified by sleep actimetry.

**Results:**

This research was funded in February 2018 and received National Health Service Research Ethics Committee approval in September 2018. The data collection period was from October 1, 2018, to November 30, 2019. A total of 24 participants were recruited for the study. The data analysis is complete, with results expected to be published before the end of 2020.

**Conclusions:**

Findings will demonstrate how often people with dementia experience binocular vision problems. If frequent, diagnosing and treating them could improve quality of life by reducing the risk of falls and fine visuomotor task impairment and by relieving psychosocial anxiety. This research will also demonstrate whether changes in depth perception, pupillary responses, and quality of vision relate to changes in memory or sleep quality and brain structure or function. If related, these quick and noninvasive eye tests help monitor dementia. This would help justify whether binocular vision and pupillary response testing should be included in dementia-friendly eye-testing guidelines.

**International Registered Report Identifier (IRRID):**

RR1-10.2196/16089

## Introduction

### What We Know About Dementia and Vision

People living with dementia (PWD) experience unique difficulties in coping with comorbidities, including visual impairment. A recent large-scale study [1] identified visual impairment as more prevalent among PWD compared with older people, generally. Overall, 19% of PWD had a remediable form of visual impairment, and other studies have identified potential links between poor visual acuity (VA) and cognitive decline [2-5]. Recent reviews highlighted that sensory deterioration such as vision and hearing loss can both contribute to or be a consequence of cognitive decline through both direct and indirect mechanisms [6]. For example, dementia-related cortical vision processing abnormalities may give rise to such a link [4,7], but visual problems have also been found to precede cognitive decline and increase the risk of dementia [5,8,9]. However, the mechanisms suggested for this association are being debated, including functional disability affecting engagement with cognitively stimulating activities [5,8,10] and interaction with depressive symptomology [8]. Overall, it is suggested that addressing global sensory deterioration may significantly impact the quality of life for PWD and their caregivers [9-11], with some studies suggesting that routine sight-restoring treatments such as cataract surgery have the potential to modify the trajectory of cognitive decline [12] or increase gray matter volume [13].

Assessment of visual function within larger studies [1,5] was limited to high-contrast VA, excluding another potential source of debilitating visual symptoms—disorders in binocular vision (BV), leading to asthenopia, strabismus, and diplopia. This, with the associated loss of depth perception, can contribute to falls in older people [14], impact fine motor task performance [15], and affect psychosocial well-being [16]. Problems with BV increase in prevalence with advancing age [17] and are often remediable with simple treatments where the etiology is related to oculomotor control, known to be disrupted in some types of dementia [7,18]. However, the literature on the prevalence of BV disorders for PWD is limited; only 3 small-sample studies have evaluated the quality of stereoacuity [2-4], with other grades of BV (eg, motor fusion) unconsidered. The findings in these studies are conflicting, potentially due to differing stereotests used between studies as the upper limit of normality [19], and the impact of refractive blur varies between stereotests [15].

### Stereopsis in People Living With Dementia

A pilot study explored the measurement of BV among people with cognitive impairment (n=7; Table 1). Although the sample size precludes a formal statistical analysis, the raw data suggest difficulty in attempting static random dot stereotests in comparison with real depth (Frisby) or dynamic (Accurate STEReotest: ASTEROID [20]) stereotests, particularly the Preschool Randot (PSR) stereotest, which lacks a monocular control to facilitate an understanding of the test.

Data were collected from council day center attendees with no history of amblyopia, strabismus, or ocular trauma, scoring ≤21 on the Mini Addenbrookes Cognitive Examination, a score highly likely to have come from someone with dementia. Near tests were conducted at 33 cm, the distance VA test was conducted at 3 m, and all stereotests were performed according to the manufacturer’s instructions. The ASTEROID stereotest was not performed in 1 patient (002) due to a history of seizures. The ethical approval reference number is 18/YH/0152.

Depth perception problems are a commonly cited aspect of living with dementia [21], and there are known impacts of Alzheimer disease on oculomotor nuclei [7], important in binocular eye coordination, and the parietal lobe, integral to binocular processing for depth perception [22]. It is reasonable to hypothesize that the strength of depth perception would deteriorate as dementia progresses, but no studies have attempted to confirm this. Visual perception problems have been proposed as a unique risk factor for falls in PWD [23,24]. Falls are more common in PWD [24], including injurious falls such as hip fractures [25], and hospitalization of older adults carries an increased risk of further cognitive decline [26]. Delirium is a common mechanism for progression of dementia following hip fracture [27]. As such, monitoring of visual functions could identify those living with dementia who are at greater risk of injurious falls and hospitalization that could exacerbate the progression of dementia. Recent reviews have called for more research into the role of visual functions as a less costly and invasive biomarker for the progression or severity of dementia [28,29]. However, the interaction among primary visual function (VA and contrast sensitivity), binocular functions such as stereopsis and horizontal fusional vergence range, and rate of cognitive decline (eg, cognitive test score) in PWD is yet to be evaluated formally. Consideration of strength of depth perception as a potential contributor to visual difficulties in dementia may therefore add predictive value to this suggested marker of disease progression.

**Table 1:** Small-sample pilot data trialed multiple stereotests with individuals who had cognitive impairment (n=7).

ID	Gender	Age (years)	Mini Addenbrookes Cognitive Examination II score	Distance acuity (logMAR)		Near prism fusion amplitude (∆ BI^a^- BO^b^)		Near heterophoria size (∆)		Frisby stereotest (arcsec)		TNO^c^ stereotest (arcsec)		Preschool Randot stereotest (arcsec)		ASTEROID stereotest (arcsec)	
				RE^d^	LE^e^												
001	F^f^	84	20	0.08	0.00		16BI-20BO		12		75		120		60		160
002	F	81	9	0.20	0.00		12BI-25BO		14		215		2480		Not measurable		Not performed
003	M^g^	82	4	0.50	0.40		16BI-40BO		8		Not measurable		Not measurable		Not measurable		1200
004	F	87	10	0.00	0.10		10BI-20BO		4		75		2480		Not measurable		570
005	M	84	13	0.15	0.45		20BI-40BO		8		40		2480		Not measurable		140
006	F	82	21	0.08	0.10		16BI-35BO		4		75		2480		400		140
007	M	70	9	0.08	0.10		6BI-16BO		20		215		2480		800		1200

^a^BI: base in.

^b^BO: base out.

^c^TNO: The Netherlands Organisation for Applied Scientific Research.

^d^RE: right eye.

^e^LE: left eye.

^f^F: female.

^g^M: male.

### Monitoring Vision to Index Dementia Progression

Emerging evidence suggests that pupil dynamics could also index the progression of dementia, potentially serving as a biomarker for early diagnosis [30]. Pupillary responses (maximum dilation velocity, average dilation, minimum and maximum size) are mediated by brainstem structures showing early pathological changes in PWD [7]. Measuring task-evoked pupil changes for PWD during a memory task (varying cognitive load level) in addition to monitoring visual, binocular, and cognitive function over time enables a comprehensive evaluation of clinical visual changes in PWD and their relationship to changes in cognitive status via indicators such as the standardized Mini Mental State Examination (MMSE) score.

The addition of structural and functional magnetic resonance imaging (MRI) would enable preliminary evaluation of potential relationships between clinical visual changes, cognitive decline, and cortical structure or activation in PWD. This can be coupled with an evaluation of sleep quality, which is an emerging area of interest in Alzheimer disease due to sleep fragmentation being identified as a risk factor for cognitive decline [31]. Anatomically, the visual system has important input into circadian rhythms [7], with cataract surgery being reported to improve sleep quality because of better light transmittance [32,33]. Furthermore, a recent cross-sectional study identified visual impairment as being associated with the presence of sleep problems in institutionalized individuals with dementia [34]. Despite the known clinical relationship between sleepiness and deterioration of binocular coordination, research in this area has focused on the effects of major or total sleep deprivation [35-37]. No study to our knowledge has attempted to formally explore the relationship between quality of sleep measures, such as sleep latency and fragmentation, and clinical measures of BV. The mapping rate of brain atrophy (on structural MRI) and blood oxygenation level dependent (BOLD) activation levels in visual areas (on functional MRI) against visual function, binocular function, rate of cognitive decline, and baseline sleep quality may help determine whether clinical visual changes possess predictive or diagnostic potential as well as identify possible mechanisms for such an interaction.

This proposed exploratory observational study attempts to address these gaps in the literature based on the study aims presented above. Performing this study on an exploratory basis enables the inclusion of multiple dementia types because of the overall dearth of literature on the occurrence of BV disorders and pupillary changes among dementia types other than Alzheimer disease. Answering these research questions will determine whether changes in BV and pupil dynamics, in addition to changes in visual function, should be given further consideration as potential markers or predictors of both changes in cognitive status (indexed by standardized MMSE, found to agree substantially with the Clinical Dementia Rating for mild-to-severe dementia [38]) and cortical changes in structure or function during the course of recently diagnosed dementia.

## Methods

### Aims

This study aims to do the following:

1. Determine the relationship between changes over time in cognitive status (standardized MMSE score) and the following measures: binocular function (stereoacuity, motor fusion, and convergence near point [NPC]), visual function (VA and contrast sensitivity), pupillometry measures (maximum dilation velocity, average dilation, minimum and maximum size), and sleep quality (Pittsburgh Sleep Quality Index).
2. For individuals with Alzheimer disease, relate changes in the above to sleep onset latency, efficiency, and fragmentation (actigraphy and sleep diary) and structural changes in brain volumes and functional changes in cortical activation patterns within visual areas for a subgroup with Alzheimer disease.
3. Establish the prevalence of BV disorders and abnormal pupillometry measures among people with recently diagnosed dementia.

### Study Design

The study is an exploratory observational study.

The inclusion criteria for all participants (maximum, n=210) included the following:

1. Confirmed diagnosis, within the last 24 months, of Alzheimer disease, vascular dementia, mixed Alzheimer and vascular dementia, Lewy body dementia, or posterior cortical atrophy.
2. Baseline corrected distance VA ≤0.300 logMAR binocularly—that is, no significant bilateral visual impairment [1] affecting the ability to fixate pupillometry targets or participate in BV tests.
3. No pathology affecting pupil function, for example, ocular trauma.
4. No history of childhood amblyopia or strabismus.
5. No history of alcohol or substance misuse.
6. No significant neurological or psychiatric history excluding dementia, for example, focal brain lesion and schizophrenia.
7. Not currently taking miotic or mydriatic medications (eg, for glaucoma).
8. If taking medication that may impair cognitive function, to have been taking this medication for at least 6 weeks at a stable dose.
9. Spectacles, if worn, are up-to-date prescriptions (within the last 2 years).
10. Fundus and media check within the last 12 months, with permitted diagnoses: no apparent defect, age-related macular degeneration, diabetic retinopathy, and cataract [1].

The inclusion criteria or the subset of PWD receiving MRI (n=30; comprising a suitable subsample for the pilot study [39]) included the following:

1. Capacity to provide informed consent, as determined by the 3-Item Capacity Questionnaire or in discussion with the direct care team (this criterion is in place for this subset of participants because of the requirement for the person with dementia to be alone in the scanner room during scanning and only be addressed by intercom).
2. No contraindications for MRI, for example, embedded metals.
3. Confirmed diagnosis, within the last 24 months, of Alzheimer disease—dementia type selected for established structural MRI disease progression markers [40].

Frontotemporal dementia was excluded because of the higher likelihood of challenging behavior being exhibited [41], presenting a risk for the lone researcher during testing. Parkinsonian dementia was also excluded as the binocular function and oculomotor deficits in Parkinson disease are well documented [42-44]. A diagnosis window of 24 months was selected over the use of a cognitive test score cap to permit heterogeneity in dementia severity across the sample to explore the prevalence of BV disorders while also minimizing skew toward people with more severe dementia, which may limit the ability to effectively evaluate changes in cognitive status over time. Such heterogeneity can occur within this window because of known diagnostic delays for dementia (18-30 months [45]).

No upper or lower age limits were applied to maximize recruitment, although it was anticipated based on previous audits of the case pool of the recruiting National Health Service (NHS) Trust that the majority of potential participants were likely to be aged 60 years. Research on the prevalence of BV disorders in association with advancing age is limited and excludes PWD, but one large sample study found a 19.1% difference in the prevalence of BV disorders between adults aged between 60 and 69 years and those aged 80 years. To minimize the possible impacts of an unrestricted age range leading to a preponderance of one particular age group affecting the anticipated prevalence of BV disorders within the sample, an average prevalence for binocular vision disorders was calculated for adults aged 60 years using the data from this study and was used to inform the choice of sample size. Previous studies [46,47] have commented on the difficulty in distinguishing visual problems arising from the presence of dementia from those arising as a consequence of advancing age, but fundus or media pathologies such as cataracts and age-related macular degeneration are known to be prevalent in this patient group [1]. Studies evaluating the relationship between the presence of these pathologies and the presence of cognitive impairment or Alzheimer disease have presented conflicting results [7,48,49]. Attempting to restrict recruitment on this basis may limit the generalizability of the study findings and reduce sample size and the ability to explore the data collected.

As the proposed study focuses on tracking clinical changes in visual, pupillary, binocular, and cognitive functions within a restricted time frame, the emphasis is less on the underlying cause of these changes (ie, dementia vs fundus or media pathology) and more on exploring the relationships between them. Thus, for this study, the inclusion of individuals with certain fundus or media pathologies per a previous large-scale study (diabetic retinopathy, cataract, and age-related macular degeneration) [1] can be permitted. This enables the possibility for a subgroup analysis while still achieving the research aims.

### Recruitment

Eligible participants, identified by their direct care team within participating NHS Trust community mental health clinics or from a database of research volunteers held by the research & development (R&D) department, will be provided with study information during their clinic appointment or by post following telephone contact if they express an interest to participate.

Sample size is justified on a pragmatic basis, balancing recruitment feasibilities with the research aims of exploring potential relationships between cognitive status and visual, binocular, and task-evoked pupil function. It is anticipated that up to 210 participants could be reasonably tested within a 10-week period, determined by funding constraints to allow completion of the study within 12 months. This assumes a 60-min consent and testing slot per participant, 7 participants per day, 3 days per week. A maximum target sample of 210 participants was permitted.

The participating NHS Trust receives approximately 1890 referrals annually for people with dementia, and diagnoses up to 160 individuals with dementia monthly, providing a large pool of potential participants commonly aged 60 years (unpublished data). Among older people aged 60 years without dementia, the average prevalence of BV disorders was 31.6% [17]. If >200 PWD are recruited, approximately 63 PWD could have reduced binocular function, suitable for exploratory analysis of BV disorders and altered pupil responses with a standard error <3.5% based on the hypothesis that PWD may have greater proportions of abnormal test results than people without dementia.

PWD eligible for MRI subgroup inclusion will be serially recruited during the initial study visit until the subgroup sample size was achieved (n=30). If withdrawing after the first scan, they will not be replaced in recruitment.

### Procedures

Informed consent will be sought from participants or a consultee process will be employed where appropriate, with the capacity to consent determined by a validated brief questionnaire [50] where indicated. Ongoing consent [51,52] will be determined on a per-visit basis. The consent form also includes provisions for the participant to express their wishes concerning participation in the study, should they experience a loss of mental capacity before the study is completed, as an adjunct to these considerations.

A case history (caregiver supported if required), including dementia type and diagnosis date, ensures eligibility criteria are met. Dementia type and diagnosis date, previous ocular history, and previous imaging findings will be corroborated using the patient’s medical records after consent is obtained.

Eligibility screening is supported by optometric information (VA, current prescription, and fundus/media check findings), obtained from the patient's optometrist, or a monocular distance VA test if the last NHS sight test was >1 year ago. If the latter satisfies the eligibility criteria, an updated sight test can be arranged by the researcher (by written agreement) or the participant or their caregiver with a provider of their choice. If eligible to participate, the researcher will contact the participant to organize the initial study visit as well as an MRI scan if eligible.

All participants will be invited to complete the Pittsburgh Sleep Quality Index at each study visit—or this can be proxy-completed by a caregiver with knowledge of the participant’s sleep patterns.

The following clinical tests will be performed at a community clinic or in the participant’s home by the researcher (registered orthoptist) at all study visits:

1. MMSE: this test was selected as a global measure of cognitive status that is highly relevant to clinical dementia care and is commonly used in memory clinics and other clinical consultations in the United Kingdom. Current research suggests weaker performance of the standardized MMSE when used to monitor individuals with early cognitive impairment; however, our participants had an existing diagnosis of dementia and will have held this for up to 2 years. This limitation was balanced against the need to seek a short, global measure already familiar to PWD and their caregivers, to keep the scope of the research broad, minimize distress during cognitive testing, and keep visit durations within an acceptable time limit as advised by patient involvement volunteers. The researcher was trained by a consultant neuropsychiatrist to conduct the test and score it
2. Monocular near VA (Sonksen logMAR).
3. Monocular distance VA (Early Treatment of Diabetic Retinopathy Study: ETDRS logMAR chart or Lea acuity paddles).
4. Binocular contrast sensitivity (Cardiff contrast test).
5. Cover test (testing distances: 33 cm and 6 m).
6. Suppression (Bagolini glasses; testing distances: 33 cm and 6 m).
7. Ocular motility.
8. Binocular function: horizontal phasic prism fusion range (PFR; testing distances: 33 cm and 6 m) and stereotests (Frisby and ASTEROID; administered on a glass-free 3D tablet). Note that test selection was informed by the raw data shown in Table 1, suggesting that individuals with cognitive impairment could perform these stereotests more easily in comparison with the static random dot TNO and Preschool Randot stereotests.
9. Near point of convergence.
10. Prism cover test (testing distances: 33 cm and 6 m).
11. Pupillometry (testing the dominant eye under standard room lighting): participants attempt the Wechsler Memory Scale-III Digit Span subtest for 3 digits (low cognitive load, 4×) and 6 digits (high cognitive load, 4×); digits presented by the computer aurally at a rate of 1 digit per second, with *Ready* and *Repeat* at start and finish; and pupil diameter and dynamics recorded when participants repeated digit sequences, using Tobii X2-60 (Tobii Ltd; 9-point calibration, dimmed lighting, 20-second calibration data stream recording followed by a total of 8 × 15-second data stream recordings—video recordings of the iris and pupil are not saved).

The testing time is approximately 60 min in total (including computer setup), with breaks offered if required. All tests are low-risk, quick, noninvasive, and (except for pupillometry) routinely performed in orthoptic practice.

All participants will return for 2 follow-up assessments, 4 months apart (plus or minus 2 weeks), repeating the above tests. Adaptations required to enable performance of a test will be documented, for example, change in test target, use of matching card, demonstration of testing procedure, and instances where performing tests was not possible.

The MRI subgroup (n=30; diagnosis of Alzheimer disease) will also receive an MRI scan (within 2 weeks of the first and final study visits) using a 3T TIM Trio MR scanner (Siemens) with a 32-channel array head coil.

Whole-brain scans performed included the following:

1. Structural MRI (11 min in total): T1-MPRAGE, FOV 192 mm, matrix 256 × 256, 1 mm isotropic spatial resolution, TR=2250 ms, TE=2.98 ms, flip angle 9°, GRAPPA factor 2, and acquisition time 4 min 32 seconds. T2-TSE, 29 × 4 mm slices, slice gap 1.2 mm, FOV 220 mm, matrix 320 × 320, 0.7 mm isotropic spatial resolution, TR=5060 ms, TE=102 ms, flip angle 140°, GRAPPA factor 2, acquisition time 1 min 38 seconds FLAIR-TSE, 25 × 4 mm slices, slice gap 1.2 mm, FOV 220 mm, matrix 256 × 256, 0.9 mm isotropic spatial resolution, TR=9000 ms, TE=100 ms, flip angle 150°, acquisition time 4 min 32 seconds.
2. Functional MRI (25 min in total): BOLD T2–EPI, 32 × 3 mm descending slices, slice gap 0.75 mm, FOV 192 mm, matrix 64 × 64, voxel size 3 × 3 × 3 mm, spatial resolution 3 mm × 3 mm, TR=2000 ms, TE=30 ms, flip angle 78°, bandwidth 2112 Hz per pixel, echo spacing 0.54 ms.

Viewing comprised the following:

1. Multifocal polar and eccentricity retinotopic mapping stimuli (4 runs of 31 × 8000 ms mini blocks alternating between 2 stimulus sets comprising sectional checkerboard wedge and ring stimuli) [53]. Acquisition time of 4 min 16 seconds per run. This paradigm was selected to enable shorter scan times and reduce the risk of fatigue while still facilitating a good signal-to-noise ratio.
2. Suprathreshold static red-green anaglyphic stereoscopic targets [22] presented using MATLAB (Matrix Laboratory; Mathworks) and Psychtoolbox-3. The target was a random dot 6 × 6 checkerboard presented against a 50% gray background, comprising interleaved black and white checks to equalize luminance across the image and ensure visibility of a consistent pattern was maintained between disparity and zero disparity viewing conditions. A checkerboard design was selected to ensure that the stimulus contained an equal number of disparity-defined and nondisparity-defined regions [54]. The black checks were disparity defined. Overall, 3 runs of 6 condition blocks and 1 control block (blank 50% contrast screen with fixation target) were performed. Each condition block sequentially displayed 6 targets, randomized for stimulus duration (2.2, 2.5, 2.9, 3.2, 3.4, and 4 seconds) and inversion (checks inverted and checks not inverted), with an interstimulus interval of 0.3 seconds. Block duration was 20 seconds, block order was randomized by drawing the test disparity in pixels from a shuffled array (control, –26, –13, 0, 0, +13, +26; minus disparities are crossed and plus disparities, uncrossed). One pixel subtended 0.031° or 111.6 arcsec at 75 cm viewing distance (projector resolution 1280 × 720, in-scanner screen size 52 cm width × 44 cm height). Thus, crossed and uncrossed disparities of 1451” arc and 2901” arc were displayed. Each run was initiated after a wait of 4× TR to allow stable magnetization to be reached. Acquisition time was 7 min. Test disparities were selected based on data [54] showing that these produced the strongest BOLD signal differences when compared to zero disparity, which is important for limiting the scan duration while still producing valid data, and verified with a pilot scan of 2 young healthy subjects without cognitive impairment. They also correlate well with our pilot data (Table 1), suggesting that people with cognitive impairment manage more gross disparities with red-green anaglyph random dot static stereotests.

The minimum duration between scans is 8 months. Visits last no more than 90 min, of which no more than 45 min will be spent in the scanner. A mounted frame and padding will be used to minimize head movement for participants. As all participants must pass a capacity assessment before entering the scanner, it is not anticipated that artifacts such as head motion are any more likely to occur than they would be for any other individual of similar age. A break will be offered between the retinotopic mapping and stereoscopic functional MRI sections to minimize fatigue. The scan order is as follows: T1, retinotopic mapping functional MRI; T2, fluid-attenuated inversion recovery; and stereoscopic target functional MRI. The researcher will speak to the participant via intercom between each of the scans to ensure that the participant is comfortable, understands what is about to happen, and is still awake.

This subgroup will also be invited to wear a sleep-tracking watch (Actiwatch, Camntech) and complete a consensus sleep diary during a 10-day period following the first and final study visits, returning the watch and diary by post in a prepaid envelope.

### Data Analysis

Abnormal test results will be determined using the criteria adapted from Leat et al (Table 2) [17].

To establish the prevalence of BV disorders, the proportion of abnormal test results will be reported and compared by age group, dementia severity, and dementia type as well as against the data in Leat et al [17]. Pupillometry measures (maximum dilation velocity, average dilation, and minimum/maximum size) for high and low cognitive loads will be similarly compared with age-related norms collected in a separate study of adults aged 50 years with a normal standardized MMSE score (>26 out of 30) using the same paradigm.

MRI data will be visually inspected for quality, and participants with excessive head motion (>3 mm in any axis or >3) will be excluded. Functional MRI data will be preprocessed and analyzed in SPM8 (Wellcome Department of Imaging Neuroscience) using the standard generalized linear model approach. Preprocessing will correct for slice timing and head motion. Smoothing will not be performed to preserve spatial resolution and, because of the within-subject design, spatial normalization will not be required. In each participant’s design matrix, the timing of the stimulus blocks will be entered as regressors of interest and convolved with the canonical hemodynamic response model; head motion parameters will be included as covariates of no interest. To analyze the multifocal retinotopic mapping results, the procedure outlined by Henriksson et al [53] will be followed: coregistration between anatomical and functional data will be performed in SPM before transfer to Freesurfer using each participant’s T1-weighted structural scans and the preprocessed functional MRI data. Eccentricity and polar angle retinotopic maps will be visualized using Freesurfer and used to denote regions of interest (ROIs) for primary visual areas V1, V2, V3, V4, and V3A. For stereoscopic image viewing, within-subject change between the first and final visits in the regional BOLD signal will be calculated for occipital and parietal lobe ROIs. Structural analyses will be conducted using Freesurfer; changes in cortical thickness in the occipital and parietal lobe ROIs and changes in the medial temporal lobe volumes will be calculated at the within-subject level.

A linear mixed model approach will assess, at the within-subject level, changes in the following variables at the level of time (visit number): pupillometry measures (time to maximum dilation, percentage maximum dilation from baseline, percentage maximum constriction from baseline, and baseline pupil size), visual function (VA and contrast sensitivity), binocular function (PSR and Frisby stereoacuity, NPC, and PFR), and standardized MMSE. Linear mixed modeling enables the consideration of repeated measures with the selection of a suitable covariance matrix. It is also effective in the presence of missing data for individual tests, making it an ideal approach to accommodate instances where PWD may, for example, be able to complete the high-load task-evoked pupillometry assessment at one visit but not the next.

Models that control for dementia type will determine relative contributions made by changes in pupillometry measures, visual function, and binocular function to changes in standardized MMSE, regional BOLD activation percentages, brain volume, and cortical thickness (first vs final visit). Results will be contrasted between PWD with and without a diagnosed BV disorder. Further subgroup analyses by presence/absence of fundus/media pathology or BV disorder will be performed depending on prevalence.

For participants who complete sleep actigraphy, linear mixed models will examine the level and rate of change in binocular function measures, standardized MMSE, regional BOLD activation percentages, brain volume, and cortical thickness (first vs final visit) as a function of sleep quality at baseline, adjusted for age, sex, and education, and their interactions with time.

**Table 2:** Criteria for abnormal test results.

Test	Abnormal result
CT^a^	Heterotropia
PCT^b^	Horizontal heterophoria >10Δ Vertical heterophoria >2Δ
PFR^c^	Horizontal: <2× heterophoria size on PCT (Δ, Sheard criterion) Vertical: >4Δ
OM^d^	Anomalous pursuit movements and/or underaction or restriction ≥–2 in any direction
NPC^e^	>10 cm
Stereopsis	Frisby: >210” arc [55] ASTEROID: ASTEROID data collected for feasibility testing only; normative data currently unavailable

^a^CT: computerized tomography.

^b^PCT: prism cover test.

^c^PFR: prism fusion range.

^d^OM: ocular motility.

^e^NPC: convergence near point.

## Results

### Current Status

This research was funded in February 2018 and received approval from the NHS Research Ethics Committee in September 2018. The data collection period was from October 1, 2018, to November 30, 2019. A total of 24 participants were recruited for the study. Data analysis is complete, and the results are expected to be published before the end of 2020.

### Anticipated Impact

Binocular, visual, and pupil function tests are routine, low-risk, quick, and noninvasive; if found to be interlinked markers of cognitive decline, monitoring their change over time could be an important aspect of future dementia care. Orthoptists, as allied health professionals, are well-placed to take on this role because of their ability to conduct objective assessments of visual function in people with cognitive difficulties, thereby obtaining robust clinical data.

If BV disorders are found to be more prevalent in this group than in older people, further research could explore the impact of treating BV disorders on quality of life for PWD and their caregivers, potentially evidencing to commissioners the need for dedicated orthoptic services for PWD. This would also provide evidence for the inclusion of assessment and treatment of BV disorders within the College of Optometrists [56] and Royal College of Ophthalmologists [57] dementia guidelines and the proposed Dementia Eye Care Pathway [58], where it does not currently feature.

## Discussion

### Ethical Considerations

#### Risks of Participation

All participants have a confirmed diagnosis of dementia as part of the inclusion criteria; thus, disclosure of diagnosis through study information materials has very low impact risk. All clinical tests used are noninvasive, quick, and low-risk, and, except for the ASTEROID stereotest and pupillometry, are routinely conducted in orthoptic practice with minimal distress to patients. The ASTEROID stereotest requires the participant to simply look at some dots on a computer screen and has been successfully performed with older people who have cognitive impairment in another study, with no distress (Table 1). Similarly, pupillometry measurement is simple and has been used extensively in people who have dementia or cognitive impairment in the past [30,59,60].

Medical imaging is a routine and well-accepted aspect of clinical care for people with dementia attending the participating NHS Trust’s community mental health clinics. Therefore, the inclusion of MRI scans for a subgroup of participants (n=30) is not anticipated to cause a significant burden. A panic button will be provided for the participant to use and their caregiver will be able to communicate with them via intercom during the scan, to provide reassurance.

If a symptomatic BV problem is diagnosed at the baseline visit, or VA deteriorates by a clinically significant amount during the study period (ie, reduction >0.200 logMAR), a report letter will be generated and sent to their general practitioner (GP) or optometrist. This allows participants, if they wish, to initiate further investigation or seek onward referral to the hospital eye service to explore treatment options. Similarly, if a gross abnormality is identified on MRI scanning, a letter will be sent to the GP, copied to their memory clinic consultant if appropriate, to prompt further investigations. This is per the standard operating procedures for the MRI facility.

Symptomatic BV disorders are associated with an increased risk of falls [14]; therefore, providing the option to seek further treatment is an important ethical consideration to mitigate this risk. As the study is exploratory and examining the relation between changes in the parameters of interest over time, rather than the etiology underlying such changes (eg, initiation of orthoptic treatment), it is still possible to include data from participants who have sought onward referral or received orthoptic treatment within analyses.

#### Financial Incentives

A nominal honorarium shows recognition and appreciation of the time and effort made by participants to attend study visits and undergo testing. As study participation involves 3 study visits and, for some participants, 2 additional visits for MRI scans, it was felt that including this honorarium was appropriate to acknowledge this burden of participation. Travel expenses are also reimbursed to ensure participants are not left out of pocket by participating in the research.

It is recognized that as part of the Health Research Authority (HRA) guidance on payments for research [61], incentives or financial inducements should not be offered where adults cannot consent. Although the research is limited to individuals with a diagnosis of dementia made in the last 24 months, there may be instances where ongoing consent changes or diagnosis is made after dementia has substantially progressed. The University of Surrey’s Research Integrity and Governance Office will also provide input and advice in these instances. Procedures regarding honorariums are explained within the study information materials.

#### Data Protection and Confidentiality

All participants will be assigned a number upon entry to the study. All paper and digital study records will use this number instead of the participant’s name to preserve anonymity. Paper project data (related to the administration of the project, for example, consent forms and record sheets) will be kept in a locked filing cabinet in a lockable room in the R&D office at the participating NHS Trust. Electronic data (primarily data sets) will be stored and pseudonymized by participant number on the university’s secure research storage server for a minimum of 10 years. Data will be disposed of securely via confidential shredding or electronic formatting after these times.

Participants will not be personally identifiable in any publication material. Participants can give permission on the consent form for their anonymous data to be deposited in a repository. Participants will be able to withdraw their data, without giving a reason, until the point at which their data is published in an anonymized form.

Anonymous data may be published with consent in data repositories (eg, Dementias Platform UK or the UK Data Archive) or as required by scientific journals to accompany article publication. These data may contain raw data or summary data (eg, means and SDs) from any measures collected. No identifiable information (eg, names, date of birth, etc) will be published. Anonymous data may be used for future research purposes by the research team or other researchers, provided the research has ethical approval.

### Patient/Public Involvement

Study information materials have been developed with 3 patient involvement representatives who have dementia or care for someone with dementia. Designing study materials with this patient group ensures that they are suitable for use in the informed consent process. This involvement work supported timely ethics review and has also assisted in developing the best method of approaching people with dementia to participate in the study.

We consulted with our involvement representatives about several issues pertaining to the study, such as the best approach toward eligibility screening and redistribution of funds allocated for honorariums where a participant does not have the capacity and is unable to receive it. We also sought advice from the patient involvement staff at the Alzheimer’s Society to ensure that our approach reflected best practices.

For honorariums, it was recommended that a discussion take place at the point of entry to the study, in instances where the participant has capacity to consent, to establish their preferences for distribution of the honorarium in the event that their capacity deteriorates. Another alternative suggested where capacity was not present at the point of enrolment in the study was reallocating the honorarium amount toward reimbursement of other costs associated with study participation as appropriate, for example, meal vouchers and increased travel budget.

For eligibility screening, our representatives shared their experiences of having their eyes tested and felt that offering to organize the test would be acceptable to avoid inducing the burden of participation, provided potential participants and their caregivers are also given the option to organize their own test if they wish and they have given consent for contact details or test results to be exchanged for this purpose.

Our patient involvement volunteers remain involved throughout the study, with additional patient involvement meetings scheduled for after data collection, to discuss any issues with the recruitment approach or study methods that may require an amendment, and at the end of the study to present the findings, establish the key take-home messages relevant to people with dementia and their caregivers, and to develop a lay summary of the findings. The Health Research Authority and INVOLVE patient involvement standards [62] were used to develop the initial recruitment materials and information pack for being involved in the study and are being used for communications with our involvement representatives throughout the study. HRA guidance on discussing public involvement embedded within the Integrated Research Application System application form was also followed.

## Abbreviations

BOLD: blood oxygenation level dependent
BV: binocular vision
GP: general practitioner
HRA: Health Research Authority
MMSE: Mini Mental State Examination
MRI: magnetic resonance imaging
NHS: National Health Service
NPC: convergence near point
PFR: prism fusion range
PSR: Preschool Randot
PWD: people living with dementia
R&D: research & development
ROIs: regions of interest
VA: visual acuity
